# Smooth Muscle Phenotype in Idiopathic Pulmonary Hypertension: Hyper-Proliferative but not Cancerous

**DOI:** 10.3390/ijms20143575

**Published:** 2019-07-22

**Authors:** Frédéric Perros, Pierre Sentenac, David Boulate, Grégoire Manaud, Tom Kotsimbos, Florence Lecerf, Lilia Lamrani, Elie Fadel, Olaf Mercier, Arturo Londono-Vallejo, Marc Humbert, Saadia Eddahibi

**Affiliations:** 1 Université Paris-Sud, Faculté de Médecine, 94270 Kremlin-Bicêtre, France; 2 Assistance Publique-Hôpitaux de Paris (AP-HP), Centre de Référence de l’Hypertension Pulmonaire Sévère, Département Hospitalo-Universitaire Thorax Innovation, Service de Pneumologie et Réanimation Respiratoire, Hôpital de Bicêtre, 94270 Le Kremlin-Bicêtre, France; 3 Unité Mixte de Recherche 999, Institut National de la Santé et de la Recherche Médicale, Université Paris-Sud, Laboratoire d’Excellence en Recherche sur le Médicament et l’Innovation Thérapeutique, 92350 Le Plessis Robinson, France; 4 Centre de Recherche de l’Institut Universitaire de Cardiologie et de Pneumologie de Québec, Laval University, Montréal, QC G1V 4G5, Canada; 5 PhyMedExp, University of Montpellier, Institut National de la Santé et de la Recherche Médicale, Centre National de la Recherche Scientifique, 34295 Montpellier, France; 6 Department of Anæsthesiology and Critical Care Medicine, Arnaud de Villeneuve Teaching Hospital, Montpellier University School of Medicine, 34295 Montpellier, France; 7 Department of Thoracic and Vascular Surgery, Marie Lannelongue Hospital, 92350 Le Plessis-Robinson, France; 8 Alfred Health, Monash University, VIC 3004 Melbourne, Australia; 9 Research Department, Marie Lannelongue Hospital, 92350 Le Plessis-Robinson, France; 10 Institut Curie, PSL Research University, Centre National de la Recherche Scientifique, Unité Mixte de Recherche 3244, Telomere and cancer lab, 75005 Paris, France

**Keywords:** idiopathic pulmonary artery hypertension, pulmonary artery smooth muscle cells, proliferation, energetic metabolism, DNA damage.

## Abstract

Idiopathic pulmonary arterial hypertension (IPAH) is a complex disease associated with vascular remodeling and a proliferative disorder in pulmonary artery smooth muscle cells (PASMCs) that has been variably described as having neoplastic features. To decode the phenotype of PASMCs in IPAH, PASMCs from explanted lungs of patients with IPAH (IPAH-PASMCs) and from controls (C-PASMCs) were cultured. The IPAH-PASMCs grew faster than the controls; however, both growth curves plateaued, suggesting contact inhibition in IPAH cells. No proliferation was seen without stimulation with exogenous growth factors, suggesting that IPAH cells are incapable of self-sufficient growth. IPAH-PASMCs were more resistant to apoptosis than C-PASMCs, consistent with the increase in the Bcl2/Bax ratio. As cell replication is governed by telomere length, these parameters were assessed jointly. Compared to C-PASMCs, IPAH-PASMCs had longer telomeres, but a limited replicative capacity. Additionally, it was noted that IPAH-PASMCs had a shift in energy production from mitochondrial oxidative phosphorylation to aerobic glycolysis. As DNA damage and genomic instability are strongly implicated in IPAH development a comparative genomic hybridization was performed on genomic DNA from PASMCs which showed multiple break-points unaffected by IPAH severity. Activation of DNA damage/repair factors (γH2AX, p53, and GADD45) in response to cisplatin was measured. All proteins showed lower phosphorylation in IPAH samples than in controls, suggesting that the cells were resistant to DNA damage. Despite the cancer-like processes that are associated with end-stage IPAH-PASMCs, we identified no evidence of self-sufficient proliferation in these cells—the defining feature of neoplasia.

## 1. Introduction

Idiopathic pulmonary arterial hypertension (IPAH), a vascular disorder associated with pulmonary circulation, is characterized by pulmonary arterial obstruction, which is mostly caused by the dominant proliferation of parietal pulmonary artery smooth muscle cells (PASMCs), as well as by variability in pulmonary vasoconstriction to a lesser extent; this leads to increased pulmonary vascular resistance, right-heart failure, and can ultimately prove fatal [1,2].

In the past decade, several major advances have led to substantial improvements in the management of IPAH. Much of the current knowledge was determined using animal models. Although none of the current animal models of pulmonary hypertension completely recapitulate the human disease, they do provide insight into the potential cellular pathways contributing to its development and progression [3]. We have learned a great deal about the pathobiology of IPAH, but we still do not know what initiates this disease and the progression of pulmonary vascular remodeling and obstruction. Although the pathogenesis and pathobiology of IPAH is complex and there is individual heterogeneity in both the clinical trajectories of patients and the inter-relationships between endothelial cell dysfunction, smooth muscle cell growth, apoptosis resistance, and a chronic inflammatory micro-environment in their distal pulmonary arteries [4,5], the most striking cellular processes underlying the observed vascular remodeling observed in this condition are related to excessive PASMC growth and apoptosis resistance. In this setting, many disease-predisposing and function-modifying characteristics of PASMCs have been identified, including oxidative stress, inflammation, and cross-talk with pulmonary endothelial cells [4,6]. Given that many of these findings overlap with cancer-like processes—especially deregulated cellular metabolism, sustained proliferation, and inhibition of apoptosis, IPAH is currently categorized by many as a cancer-like disease [7,8,9]. Although the neoplastic-like pathobiology of IPAH was originally put forward because of the finding that in IPAH, endothelial cells from plexiform lesions showed cancer-associated genetic alterations including microsatellite instability, DNA damage [10], and concomitant dysregulation of the growth–apoptosis balance [11], the “cancer-like” idea quickly spread to include the hyper-proliferating PASMCs. From this idea is born the hypothesis that the repurposing of anti-cancer drugs could constitute an effective antiproliferative/proapoptotic strategy to reverse established pulmonary vascular remodeling [12]. Accordingly, imatinib mesylate, a receptor tyrosine kinase inhibitor (TKI) that has been approved for several oncology conditions, has already been investigated for the treatment of PAH [13]. However, imatinib was poorly tolerated in some patients and although it improved cardiac output, it afforded only modest improvements in pulmonary artery pressure [14]. Conversely, dasatinib, another TKI used as an alternative to imatinib in the treatment of chronic myelogenous leukemia, has even been shown to induce PAH [15]. Consequently, it is important to identify the nature of IPAH-PASMC: cancerous or non-cancerous, to avoid wrong therapeutic strategy development and potential severe side effects of anti-cancer drugs in frail PAH patients.

The defining features of a cancer cell is self-sufficient growth, replicative immortality, and “unchecked” tissue invasion and its hallmark phenotype includes DNA damage, genomic instability, apoptosis blocking signals, and sustained angiogenesis [16].

In this study, we investigated the tumor-like phenotype of PASMCs from patients with end-stage IPAH with a major focus on self-sufficient proliferation, apoptosis resistance, replicative potential, and DNA damage/genomic instability.

## 2. Results

### 2.1. Pulmonary Artery Smooth Muscle Cells (PASMCs) from Idiopathic Pulmonary Arterial Hypertension (IPAH) Proliferate Faster than Cells from Controls

**Figure 1:**
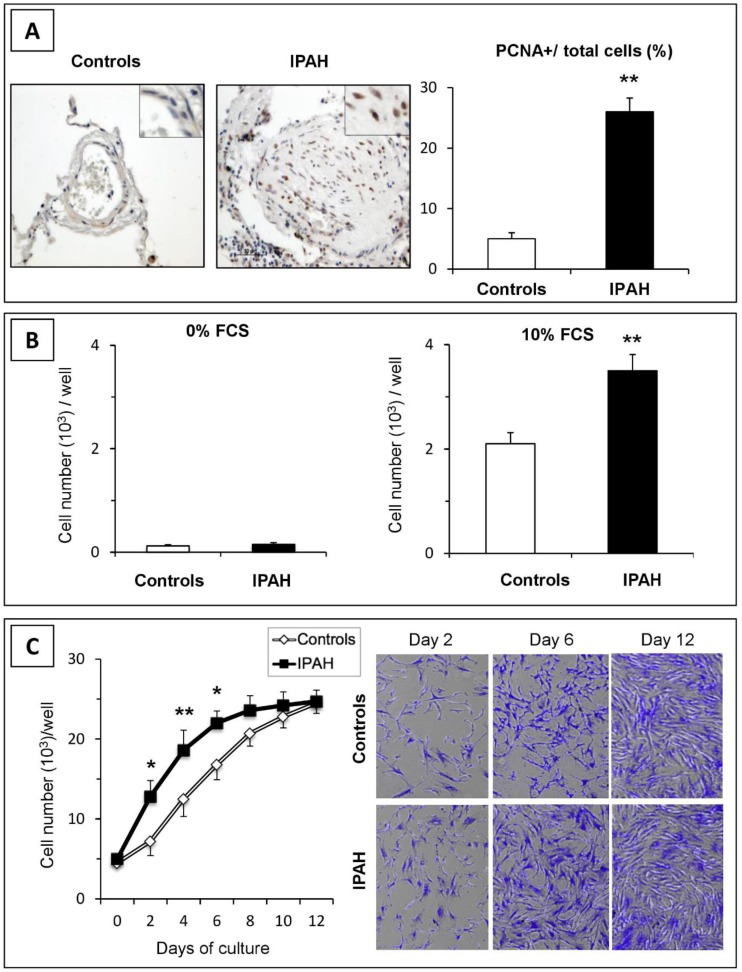
Proliferation of pulmonary vascular cells in idiopathic pulmonary artery hypertension (IPAH). (**A**) In situ cell proliferation in pulmonary arteries from control and IPAH samples was estimated by immunohistochemistry for proliferating cell nuclear antigen (PCNA). Positive cells were more numerous in pulmonary arteries from patients with IPAH than those from controls. (**B**) In vitro proliferation of pulmonary artery smooth muscle cells (PASMCs) cultured without or with 10% FCS for 48 h. (**C**) Cellular growth curves. IPAH-PASMCs grew faster during the exponential phase of growth, but reached a similar density at confluence compared to controls (*n* = 6 in each group). * *p* < 0.05; ** *p* < 0.01.

The proliferation status of PASMCs was first measured in situ by proliferating cell nuclear antigen (PCNA)-immunodetection in lung tissues from control and IPAH patients (Figure 1A). As shown, the number of PASMC nuclei positive for PCNA was higher in the distal pulmonary arteries of patients with IPAH than in controls (*p* < 0.01). Furthermore, the increased growth of IPAH-PASMCs was observed in vitro (Figure 1B). Cultured IPAH-PASMCs grew faster than control (C)-PASMCs in the presence of fetal calf serum (FCS) (*p* < 0.01). However, no proliferation was observed in the absence of the exogenous growth factor. These results demonstrate that although PASMCs from IPAH patients proliferate faster than those from controls, they do not display self-sufficient proliferation, as do bona fide cancer cells (Figure S1).

### 2.2. IPAH-PASMCs and C-PASMCs Respond to Contact Inhibition

Once the PASMCs were seeded, their proliferative capacity in response to FCS was analyzed. For each sample, we estimated the cell number by counting and through crystal violet staining. As shown in Figure 1C, the IPAH-PASMCs and C-PASMCs grew exponentially over the first several days. Although the proliferation rate of IPAH-PASMCs was initially higher than that of controls, both growth curves plateaued at similar stages, suggesting that cell contact growth inhibition mechanisms worked equally efficiently in both groups.

### 2.3. IPAH-PASMCs Display Consistent Proliferation Potential and Telomere Length Stability, But Are Not Immortal

**Figure 2:**
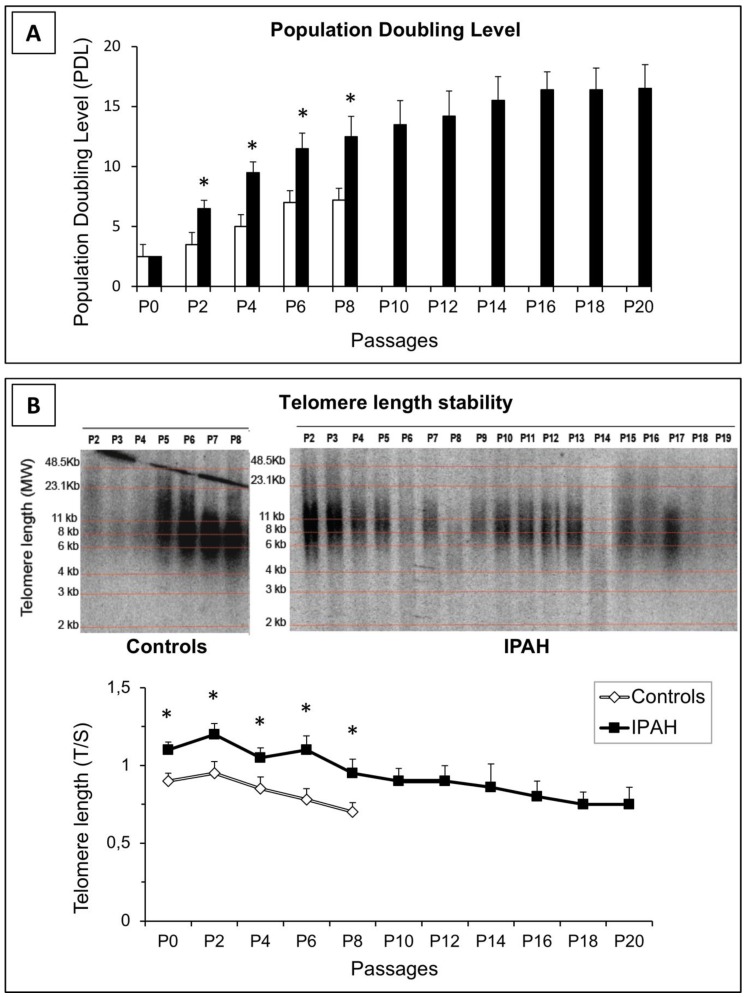
Growth capacity of PASMCs. (**A**) Population doubling level (PDL) was calculated at each passage by cell counting. In contrast to control cells, IPAH-PASMCs continued proliferating for many more passages. * *p* < 0.01. (**B**) Changes in telomere length in control and IPAH-PASMCs with advancing population doubling in vitro, as illustrated by Southern blotting (representative image) and qPCR (bottom panel). * *p* < 0.01 between controls and IPAH.

To measure the proliferation potential of PASMCs, cells from IPAH patients and controls were passaged in culture and the population doubling level (PDL) was determined. As shown in Figure 2A, PASMCs from controls did not exceed 9–10 PDL, whereas PASMCs from IPAH patients reached 16–17 PDL. However, no further proliferation was recorded for these cells even after prolonged culture (1–2 weeks), indicating that IPAH-PASMCs do not display indefinite proliferation capacity. Moreover, the estimated telomere length in C- and IPAH-PASMCs after several passages revealed that telomere length was consistently greater in IPAH cells than in controls; however, telomere length was found to decline gradually as the PDL increased (Figure 2B). All this suggests an initially “adaptive” response that then wanes.

### 2.4. IPAH-PASMCs are More Resistant to Apoptosis

**Figure 3:**
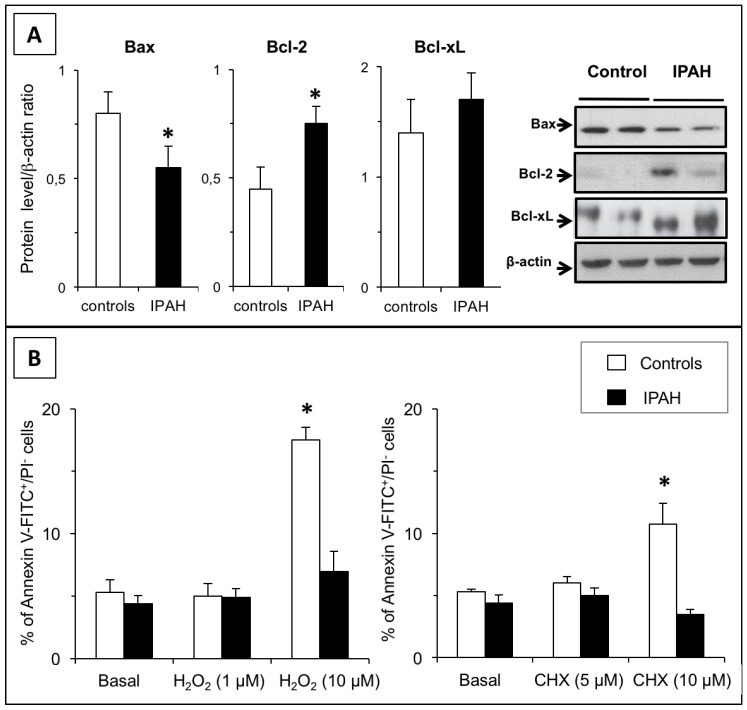
Apoptosis resistance in IPAH. (**A**) Expression levels of pro- and anti-apoptotic proteins Bax, Bcl-2, and Bcl-xL in PASMCs from controls and from patients with IPAH (*n* = 6 in each group). (**B**) The effect of H_2_O_2_ (1–10 µM) or cycloheximide (5–10 µM) on apoptosis, as determined by annexin V staining. As shown, the two treatments induced apoptosis in control (**C**)-PASMCs, while IPAH-PASMCs remained unresponsive. * *p* < 0.01 between controls and IPAH.

Initially, we checked the expression levels of key pro- and anti-apoptotic factors. As shown in Figure 3A, the IPAH phenotype was associated with decreased Bax and increased Bcl2 protein levels compared to those in cells from control subjects, while the protein levels of Bcl-xL, a member of the Bcl-2 family, were unaltered.

We next evaluated the apoptotic response to stress in PASMCs. Both hydrogen peroxide and cycloheximide strongly induced apoptosis in C-PASMCs (Figure 3B). However, no changes in the annexin V-positive cell count was observed in IPAH-PASMCs, indicating that they were more resistant to apoptosis.

### 2.5. Mitochondrial Function and Biogenesis in IPAH-PASMCs

**Figure 4:**
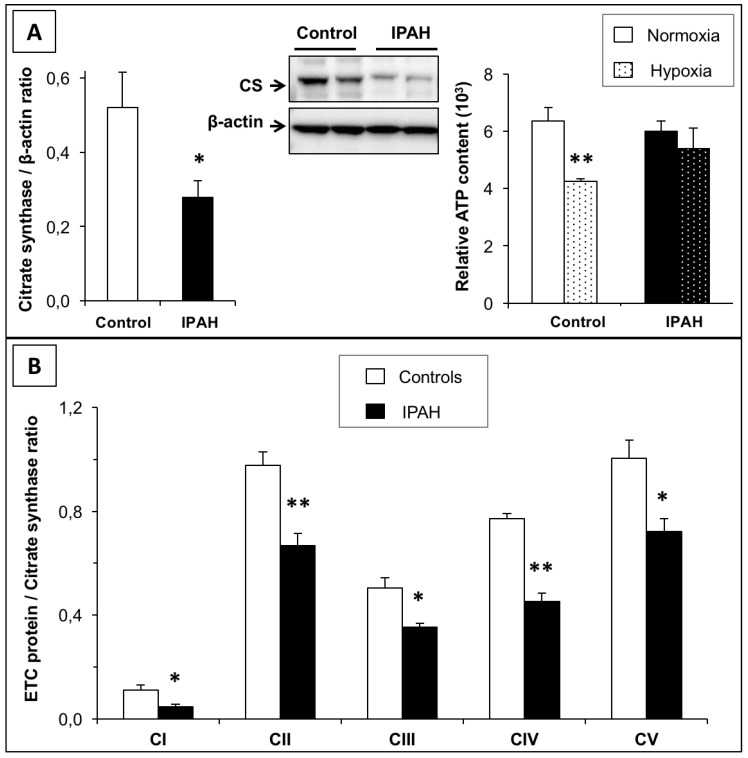
Energetic metabolism shift from mitochondrial respiration to glycolysis in IPAH. (**A**) Citrate synthase protein expression was lower in IPAH-PASMCs than in C-PASMCs. Measurement of ATP content in C-PASMCs and IPAH-PASMCs maintained for 24 h in normoxia (21% O_2_) or hypoxia (2% O_2_) (n = 5 in each group). (**B**): The levels of mitochondrial electron transport chain components were measured. The levels of different proteins were normalized against citrate synthase. * *p* < 0.05 and ** *p* < 0.01 between controls and IPAH.

Citrate synthase expression/activity, the electron transport chain, and ATP synthesis are common markers of mitochondrial function and play key roles in aerobic respiration. Previous studies [17,18] have identified site-specific defects in the electron transport chain in patients with IPAH. This finding was confirmed in the present study by measuring changes in citrate synthase (CS) protein levels, which were markedly reduced in IPAH-PASMCs compared to control cells (Figure 4A). Interestingly, as shown in Figure 4B, the expression of mitochondrial electron transport chain components was significantly reduced in IPAH cells compared to controls, supporting our hypothesis that mitochondrial respiration levels decreased in IPAH-PASMCs. Therefore, we reasoned that IPAH-PASMCs might display reduced energy production. To test this hypothesis, the ATP content in the two cell types was quantified (Figure 4A). Under normoxia, the ATP content in IPAH-PASMCs cells was similar to that in controls. However, in a hypoxic environment, ATP in control cells dropped by 30%. Strikingly, IPAH-PASMCs exhibited similar levels of ATP content under both conditions. This suggests that IPAH-PASMCs have a reduced dependence on cellular respiration for energy compared to control cells; this dependence is related to a metabolic shift to aerobic glycolysis.

### 2.6. Genomic Stability in IPAH-PASMCs

**Figure 5:**
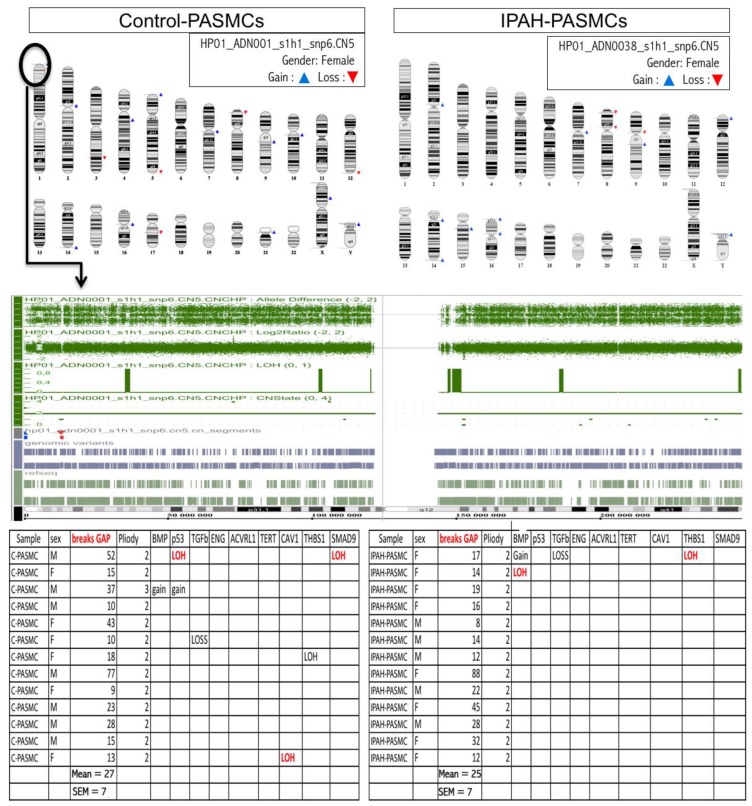
Genomic stability in IPAH. (**Upper panel**) Representative comparative genomic hybridization (CGH) array showing the gain, loss, and location of DNA regions on each chromosome. (**Lower panel**) The number of break-points in ten controls (C-PASMCs) and ten patients with IPAH (IPAH-PASMCs) as well as the gain, loss, and loss of heterozygosity (LOH) in all genomic DNA regions; this was done by focusing on regions encoding genes identified to be involved in the development of IPAH.

Array comparative genomic hybridization (CGH) was performed on genomic DNA from cultured PASMCs (Figure 5). Multiple random breakpoints were detected in both groups, but the average number did not differ between groups (27 ± 7 vs. 25 ± 7 breakpoints in controls and IPAH, respectively). Moreover, multiple regions not showing recurrent gains, losses, and loss of heterozygosity (LOH) were identified, but the event levels did not differ between the two groups; we did not identify any major aberrations in regions encoding key genes related to IPAH susceptibility or oncogene tumor suppressor genes. Genomic DNA alterations were found at similar levels in IPAH and controls and seemed to be arbitrary phenomena rather than disease-associated. We can therefore conclude that genomic instability is not a hallmark of IPAH-PASMCs.

### 2.7. DNA Damage Markers in IPAH-PASMCs

**Figure 6:**
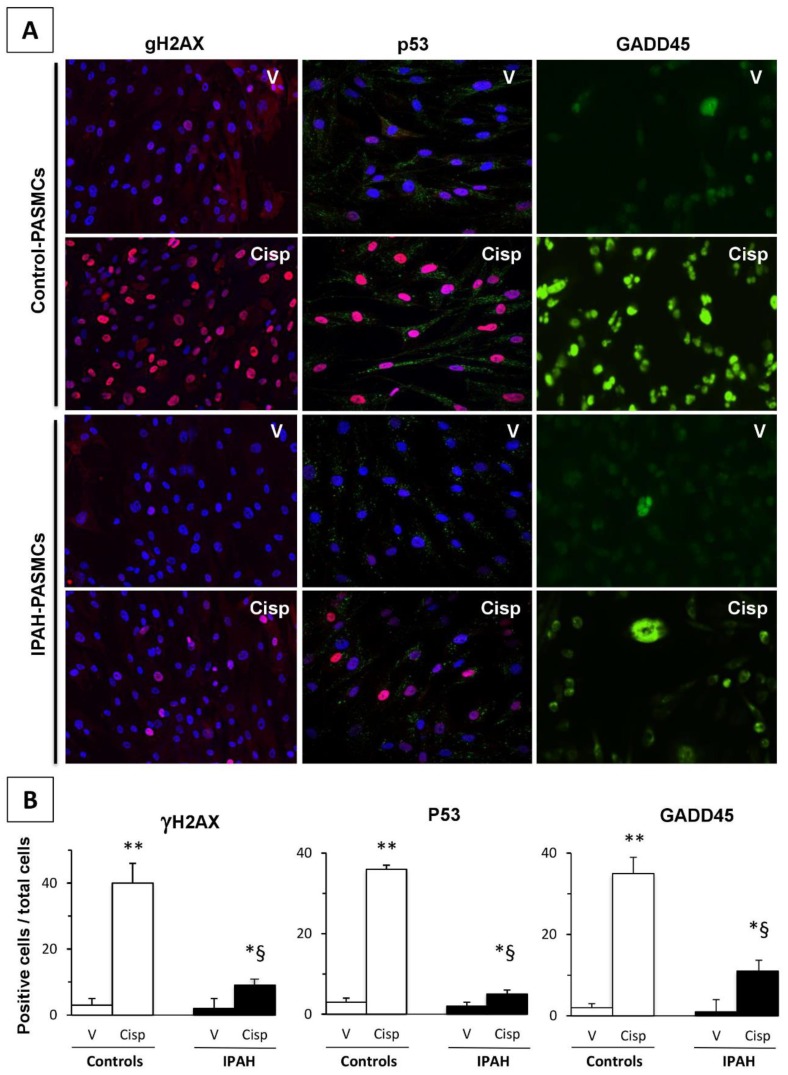
DNA damage and repair in IPAH. (**A**) Representative fluorescence microscopy images of phosphorylated γH2AX, p53, and GADD45—factors required for cell cycle arrest. Stimulation with cisplatin caused a sharp increase in γH2AX phosphorylation, which was higher in controls than in IPAH samples. Phospho-p53 and - growth arrest and DNA damage-inducible (GADD)45 staining patterns also showed similar profiles. (**B**) The ratio of positive cells/total cells in each condition was quantified in PASMCs (*n* = 6 in each group). 100× magnification. * *p* < 0.05, ** *p* < 0.01 between vehicle and cisplatin. § *p* < 0.01 between control- and IPAH-PASMCs under the same conditions.

Here, we investigated the expression of key proteins related to DNA damage repair. The measurements were performed under basic conditions and in response to genotoxic stresses induced by cisplatin. The proteins analyzed included γH2AX, p53, and its target GADD45. As illustrated in Figure 6A, under basic conditions, the signal intensity of all proteins studied was low but different between control and IPAH-PASMCs. Moreover, cisplatin treatment induced the accumulation of phosphorylated γH2AX, p53, and growth arrest and DNA damage-inducible (GADD)45; this increase in phosphorylation was more marked in controls than in IPAH-PASMCs (Figure 6B).

## 3. Discussion

The aim of this work was to characterize the phenotype of IPAH-PASMCs from end-stage IPAH patients requiring lung transplantation, and in particular to compare their phenotypes with those hallmarks of tumor cells phenotypes. Our results indicate that although the phenotype of IPAH-PASMCs was clearly different from that of healthy cells these differences did not reach the generally accepted thresholds required to categorize these cells as neoplastic. More particularly, our findings revealed a clear propensity for IPAH-PASMCs to be hyper-proliferative: firstly, the remodeling of pulmonary arteries was intimately associated with the increased growth of PASMCs; secondly, this phenomenon was also maintained in isolated and cultured cells in vitro; and thirdly, IPAH-PASMCs showed faster growth than control cells in response to FCS. However, critically we did not observe any proliferation in the absence of exogenous stimulation. Taken together, these data suggest that IPAH-PASMCs did not display self-sufficiency in growth signals, a defining characteristic of tumor cells. Nevertheless, the expression profile of IPAH-PASMCs was abnormal, and although *BMPR2* mutation and surviving overexpression may result in increased growth and apoptosis resistance in these cells, this was shown to be insufficient to induce proliferation independent of environment. IPAH-PASMCs proliferated faster and for longer periods than C-PASMCs, but this phenomenon was clearly limited in a temporal and spatial manner. Indeed, once cells reached confluency, their growth slowed dramatically. The observed growth rate of IPAH-PASMCs was consistent with the features of apoptosis resistance and indeed the aberrant protein levels of Bax and Bcl2 may have contributed to the promotion of apoptosis resistance.

According to some studies, the similarities between IPAH and cancer [12] are based on observed abnormalities in aerobic glycolysis in both conditions, known as the Warburg effect [19]. The glycolytic shift may underlie the resistance to apoptosis and increased vascular cell proliferation [20]. It may also represent an adaptive response to a locally hypoxic environment. Our results confirmed these findings in that IPAH-PASMCs showed a decrease in citrate synthase expression and electron transport chain content in contrast to control PASMCs and ATP synthesis in IPAH-PASMCs was relatively unaffected by exposure to hypoxia. Although our results support a metabolic shift to aerobic glycolysis with an overexpression of glucose transporter Glut1 [21] (Figure S2), we would emphasize that the Warburg effect is not exclusive to a cancer cell phenotype as it has also been observed in other “stressed tissue micro-environments” represented by chronic diseases that include tuberculosis, idiopathic pulmonary fibrosis [22], atherosclerosis [23], and Alzheimer’s disease [24].

The replicative capacity of the cells, as estimated by our doubling-population curve, showed that even though the IPAH-PASMC populations were maintained over time compared to control cells, this abnormality remained limited and did not extend beyond twenty passages; thereby precluding IPAH-PASMC immortalization. The relationship between telomere shortening and cell longevity has been clearly established and was another focus of our phenotypic characterization of “end-stage” IPAH-PASMCs [25]. In tumor cells, telomere loss can be prevented by upregulating telomerase expression, which elongates short telomeres and allows for continuous growth [26]. In our work, despite the observation that telomere length was greater in IPAH cells than in controls, telomere shortening with advancing culture passages was observed in both groups. This difference could not be attributed to the ages of the patients [27], suggesting that the telomere stability seen in our IPAH patients was not confounded by younger patient age.

The DNA double-strand break (DSB) is a serious lesion that can initiate genomic instability, ultimately leading to cancer [28]. Previous studies have associated the growth of pulmonary vascular cells with genomic instability. These alterations have been widely studied and validated in the endothelial cells that form plexiform lesions in IPAH [11], but this question has remained unsolved in smooth muscle cells. Array-based CGH is a powerful tool for the detection of chromosomal abnormalities in various diseases [29,30]. Array CGH performed on genomic DNA from control- and IPAH-PASMCs demonstrated a similar number of chromosomal break-points, with no significant loss or gain in any specific region. These results show that the genomes of IPAH-PASMCs are remarkably stable. Therefore, the abnormal phenotype of IPAH-PASMC is not associated with genomic aberrations and therefore unlikely to be causally linked to this pathology. Furthermore, telomere length was found to be increased in IPAH-PASMCs and it is known that functional telomeres are a major factor in genome stability [31]. Hence, although Yeager et al. [11] have previously described genomic instability in endothelial cells from plexiform lesions, our results did not support an extension of these alterations to PASMCS. Additionally, we note that Aldred et al. [32] have previously identified genomic instability in vascular cells, as well as in bronchial and circulating cells, from various conditions suggesting that genomic alterations are not a specific feature of any one disease process.

Finally, we cannot assess genomic stability without also investigating DNA damage and repair processes [33]. DSBs are the most severe deleterious DNA lesions, and display severe consequences on genomic stability and cell viability. γH2AX phosphorylation is considered a quantitative marker of DSBs [34]. As illustrated in our study, the accumulation of γH2AX in response to cisplatin stimulation was more marked in controls than in IPAH-PASMCs. Furthermore, a weak signal was observed in cells maintained without genotoxic stresses. Interestingly, the signal was more pronounced in controls than in IPAH-PASMCs upon exposure to DNA damage inducers such as ROS and inflammatory signals. Cells respond to DNA damage by activating the DNA damage response (DDR) pathway, which is orchestrated by p53 [35]. Similar to γH2AX, the nuclear accumulation of phosphorylated p53 and its target GADD45 [36] was more marked in controls than in IPAH-PASMCs after genotoxic stress. This suggests that the abnormal micro-environment had induced a “protective” adaptive response in the IPAH-PASMCs that was maintained until end-stage disease. DSB signaling and γH2AX accumulation were also associated with apoptosis, replicative senescence, or cell cycle arrest. These data further confirm that IPAH-PASMCs are resistant to apoptosis. Although DDR is a complex network comprised of many DDR factors and cell cycle regulators, the DDR was found to preferentially converge on p53, leading to cycle arrest and apoptosis, both of which require GADD45 activity. If we consider γH2AX as a faithful marker of DNA damage, it follows that IPAH-PASMCs are more resistant to DNA damage related to both endogenous (inflammatory factors, ROS, etc.) and exogenous stresses (cisplatin). It is difficult to identify the key element responsible for this phenotype, but we can hypothesize that this phenotype is a consequence of major adaptations by the cells to a stressful environment, including telomere length maintenance, relative genomic stability, and probable changes in epigenetic status.

Overall, our current work questions many studies that show almost overlapping molecular and cellular mechanisms between cancer and PAH vascular cells [12]. Those studies support the targeting of signaling pathways involved in cancer biology for controlling/regressing enhanced proliferation, survival, and apoptosis resistance of lung vascular cells in PAH. Accordingly, many anti-cancer drugs have been effective in attenuating/reversing experimental pulmonary hypertension in rat or mouse [12]. One of them, imatinib, has been presented as an anti-remodeling agent in this disease, and showed promise as a potential treatment for pulmonary hypertension in preclinical models [37] but there is no evidence of such effects in humans [13]. The reversal of pulmonary vascular remodeling should lead to time-dependent reductions in pulmonary pressures, yet neither long-term randomized controlled trials nor their respective extension phases have reported this phenomenon in a convincing way [14]. In addition, the clinical effects on exercise capacity (six-minute walk test distance) were conferred within the first weeks of therapy, and this improvement shortly thereafter reached a plateau when no further improvements are observed [13], which indicates either that the proposed anti-remodeling effects of imatinib in PAH were an oversimplification of a much more complex process or that targeting proliferation in PAH results in a rather limited treatment effect. Accordingly, we identified no evidence of self-sufficient proliferation in IPAH-PASMC, despite a previous report stating that these cells do proliferate in serum-free media [38]. In this study, IPAH-PASMC had yet a glycolytically active proliferative cell phenotype with elevated ATP generation in both serum-replete and serum-deplete conditions, that we could not detect in our culture cell conditions. However, primary cancer cells also frequently “stop” proliferating after several passages as they undergo senescence. Yet, when left in culture, some of these cells will restart proliferation after several weeks, which is when they usually have become immortal. In our study, we stopped culturing PASMC when cells became senescent. The resistance of IPAH-PASMC to DNA damage that we observed in our study also challenges the popular idea that DNA damage signaling pathway is important for PAH development [10]. However, this limitation should be nuanced by the fact that crucial pathophysiological differences exist between endothelial and smooth muscle cells. Indeed, while it seems accepted that pulmonary microvascular endothelial cells and bone morphogenetic protein receptor 2 (*BMPR2*)-deficient ECs derived from patients with IPAH are more vulnerable to DNA damage [39,40], it has been demonstrated that this susceptibility is not observed with identical genotoxic stresses in PASMCs [40]. This is supported by the work of Aldred and coworkers, who showed that somatic chromosomal abnormalities were predominantly found in PAH-ECs and not in PAH-PASMCs [32].

## 4. Material and Methods

### 4.1. Patients

This study was approved by the local institutional review board and by the local ethics committee (Comité de Protection des Personnes, Ile-de-France VII, Le Kremlin-Bicêtre, France, N° CO-08-003, ID RCB 2008-A00485-50, approved on June 18th 2008). Written informed consent was obtained from all patients prior to experimentation. For in vitro studies, we used lung specimens obtained during lung transplantations in patients with IPAH, or during lobectomy or pneumonectomy procedures for patients with localized lung cancer as controls. The average age (mean ± SD) was 38 ± 10 years in patients with IPAH and 52 ± 12 years in the controls. The mean pulmonary artery pressure (PAP) in patients with IPAH was 64 ± 17 mmHg. Preoperative echocardiography was performed on the controls to rule out pulmonary hypertension, and lung specimens from the controls were collected at a distance from tumor foci.

### 4.2. In Situ Evaluation of PASMC Proliferation

To assess in situ vascular cell proliferation in patients with IPAH, proliferating cell nuclear antigen (PCNA) staining was performed. Tissue sections were deparaffinized in toluene, treated with a graded series of ethanol washes, and rehydrated in PBS (Sigma Aldrich, St. Louis, MO, USA). Slides were then incubated for 30 min in a protein blocking solution (10% goat serum in PBS) and incubated for 1 h with an anti-PCNA mouse monoclonal antibody (M0879, clone PC-10, 1:200; Dako, Santa Clara, CA, USA) in the presence of streptavidin/biotin endogenous blocking reagents (SP-2002; Vector, Burlingame, USA). The slides were then incubated with a mouse biotinylated secondary antibody for 30 min, followed by amplification with the Vectastain ABC-AP Kit (AK-5002; Vector) for 30 min. The slides were then processed using the Vector Red Alkaline Phosphatase Substrate Kit (SK-5100; Vector).

### 4.3. Measurement of In Vitro Cell Growth

Human PASMCs were cultured from the explants of pulmonary arteries derived from patients who received transplants for IPAH and from controls. PASMCs were cultured in 10% FCS/DMEM as previously described and used between passage 4 to 6 unless otherwise specified (Figure 2) [41]. Immunofluorescence microscopy revealed stable expression of the smooth muscle cell marker SM22 by cultured control and IPAH PASMCs from early (P3) to late (P9) passages (Figure S3). Cells were seeded in 6-well plates in 10% FCS/DMEM at a density of 5 × 10^4^ cells/well and allowed to adhere for 24 h. The medium was subsequently removed, and the cells were subjected to growth arrest by incubation with serum-free DMEM. After 48 h, the medium was replaced with fresh DMEM supplemented with 10% FCS and the cells were incubated for 2, 4, 6, 8, or 12 days. At the end of each incubation period, the cells were dissociated using trypsin and counted with a Muse Cell Analyzer (Darmstadt, Germany) according to the manufacturer’s instructions. The same cells were subjected to crystal violet staining to examine their survival rate and density as previously described [42].

### 4.4. Cell Apoptosis Measurement

Flow cytometry was used to detect the rate of apoptosis in PASMCs. Cells were treated with H_2_O_2_ (1 and 10 µM) or cycloheximide (CHX, 5 and 10 µM) for 24 h, then dissociated using trypsin and washed twice with PBS. Cells were prepared as a single-cell suspension at 1 × 10^6^ cells/mL and resuspended in 100 μL annexin V-FITC binding buffer, 5 µL propidium iodide [24], or both, in the dark for 30 min at room temperature; thereafter, 400 µL binding buffer was added to wash the annexin/PI stained cells. Untreated cells served as the negative control. Apoptotic cells were then analyzed using a flow cytometer (Becton Dickinson, Franklin Lakes, NJ, USA). The rate of apoptosis was calculated as the ratio of the number of annexin V-positive cells to the number of PI-negative cells.

### 4.5. Western Blotting

Control and IPAH cells were lysed in a buffer containing 20 mM Tris (pH 7.5), 150 mM NaCl, 1 mM EDTA, 1 mM EGTA, 1% Triton X-100, 2.5 mM sodium pyrophosphate, 1 mM β-glycerolphosphate, 1 mM Na_3_VO_4_, 1 μg/mL leupeptin, and 1 mM phenylmethylsulfonyl fluoride. The protein concentration was determined using a Bradford protein assay (Bio-Rad Laboratories, Hercules, CA, USA). Samples containing 30 μg protein were separated by 10% SDS-PAGE and transferred to nitrocellulose membranes. Proteins related to cell apoptosis (Bax, Bcl2, and Bcl-xL) were detected using specific antibodies. Citrate synthase and mitochondrial electron transport chain components were then detected using a MitoProfile Total OXPHOS antibody cocktail (ab110413, Abcam, Cambridge, UK). Next, the corresponding secondary antibodies were added at a dilution of 1:10000 (Calbiochem, San Diego, CA, USA). Immunoreactive bands were visualized using enhanced chemiluminescence (ECL; GE Healthcare, Little Chalfont, UK) on a Bio-Rad Fluoro-S-Max Chemidoc system. A polyclonal antibody against β-actin (1:3000 dilution; Sigma Aldrich, St. Louis, MO, USA) served as an internal loading control. Densitometric quantification of the immunoblot bands was performed using Bio-Rad Quantity One software.

### 4.6. In Vitro Lifespan Measured Based on Population Doubling Level

In culture, an untransformed cell line has a finite lifespan that can be expressed in terms of the population doubling level (PDL). This measurement describes the total number of times the cells in the population have doubled in vitro since primary isolation. At each sub-cultivation, confluent PASMCs were trypsinized, counted, and reseeded at a density of 1 × 10^5^ cells per dish. The PDL at each sub-cultivation was calculated based on the cell count.

### 4.7. Telomere Length Measurement

We measured the changes in telomere length as the cells divided. At each passage, PASMCs were harvested and used for genomic DNA extraction with the QIAamp DNA kit (Qiagen, Hilden, Germany). DNA integrity was evaluated by SYBER Green I staining after electrophoresis on a 1% agarose gel. DNA was digested with HinfI (10 U) and RsaI (10 U) (Roche, Basel, Switzerland) and restriction fragments were analyzed by Southern blotting as described previously [43]. For telomere length measurement, qPCR was performed as previously described by Cawthon [44]; the ratio of telomere repeat copy number to single-gene copy number (T/S) was measured by comparison with the *36B4* gene.

### 4.8. Comparative Genomic Hybridization Analysis

The genomic DNA of PASMCs from control (C-PASMCs) and IPAH (IPAH-PASMCs) patients (*n* = 10 in each group) was extracted. Following the denaturation of probe DNA, hybridization was carried out using Affymetrix cytogenetic microarrays (Affymetrix Inc., Santa Clara, CA, USA) according to the manufacturer’s instructions. This technique is based on the competitive hybridization of control and patient DNA samples to an immobilized target sequence on a glass slide. Recently, array comparative genome hybridization (CGH) has proven to be a powerful tool for the detection of submicroscopic chromosome abnormalities in various diseases. The analysis allows for the detection of breakpoints and for the assignment of a status to each chromosomal region giving the gain, loss, and loss of heterozygosity (LOH).

### 4.9. ATP Content Measurement in C- and IPAH-PASMCs

ATP in cells was measured under normoxic (21% O_2_) or hypoxic (2% O_2_) conditions using a luciferase-based luminescence assay kit (PerkinElmer, Waltham, MA, USA). Briefly, cells were plated in 24-well plates at 10,000 cells/well for attachment overnight. Then, cells were exposed to normoxia or hypoxia for 4 h. Thereafter, an equal volume of the single-one-step reagent provided by the kit was added to each well and incubated for 15 min with shaking at room temperature. Cellular ATP content was measured using a luminescent plate reader. An additional plate with the same setup was used for cell counting by hemocytometry to normalize the ATP levels to the cell number.

### 4.10. DNA Damage Response Analysis

PASMCs from controls and IPAH patents were seeded on glass slides, grown for at least 48 h, and then treated with cisplatin (10 µM) or vehicle for 2 h. Cells were permeabilized with buffer containing 0.5% Triton-X, 20 mM HEPES, 50 mM NaCl, 3 mM KCl, followed by fixation with 2% paraformaldehyde at room temperature for 20 min and 100% methanol at −20 °C for 10 min. Cells were exposed to anti-phospho-γH2AX (mouse monoclonal antibody; Abcam, Cambridge, UK), anti-phospho-p53 (rabbit polyclonal antibody) and anti-growth arrest and DNA damage-inducible 45 (GADD45; rabbit polyclonal antibody; Cell Signaling Technology, Danvers, MA, USA) at 1:200 dilution in 2% BSA and 0.1% Triton X-100, followed by incubation with fluorescent secondary antibodies (Alexa Fluor-488 goat anti-mouse and Alexa Fluor-568 goat anti-rabbit; Invitrogen, Carlsbad, CA, USA) at 1:1000 dilution and counterstaining with DAPI. The slides were analyzed with an epifluorescence microscope and NiS element software (Nikon 80i, Tokyo, Japan) and the number of positive cells per field was counted.

### 4.11. Statistical Analyses

All data are reported as mean ± SEM. To assess the differences in cell proliferation between PASMCs from patients and control subjects, analysis of variance (one-way ANOVA) was used for comparison between groups. When ANOVA indicated significance and an interaction, a nonparametric Mann–Whitney test was used to compare patients and control subjects. Tukey’s post hoc test was performed to evaluate differences in ATP synthesis under normoxia and hypoxia, as well as the response to cisplatin vs. vehicle in C-PASMCs and IPAH-PASMCs. Matched ANOVA or paired *t*-tests were used when values are paired. Values of *p* < 0.05 were considered to indicate statistically significant differences between groups.

## 5. Conclusions

Both experimental and clinical data have led many towards a seductive comparison between IPAH and cancer. Our findings clearly indicate the although IPAH-PASMCs are hyper-proliferative they do not fulfill the defining phenotypic features of a cancer cell and indeed, many of the phenotypic features that we have described in the end-stage disease setting are more likely to represent ongoing adaptive changes to an abnormal micro-environment rather than maladaptive neoplastic change. Accordingly, our study does not support the direct translation of cancer biology to PAH pathophysiology.

## Supplementary Materials

Supplementary materials can be found at https://www.mdpi.com/1422-0067/20/14/3575/s1. Figure S1: Cancer cells display self-sufficient proliferation. Figure S2: Overexpression of glucose transporter Glut1 in IPAH-PASMC. Figure S3: Immunofluorescence microscopy revealed stable expression of the smooth muscle cell marker SM22 by cultured control and IPAH PASMCs from early (P3) to late (P8) passages.

## Abbreviations

PASMC	pulmonary artery smooth muscle cell
PCNA	proliferating cell nuclear antigen
IPAH	idiopathic pulmonary artery hypertension
PCNA	proliferating cell nuclear antigen
DSB	double-strand break
PDL	population doubling level
CGH	comparative genomic hybridization
CHX	cycloheximide
LOH	loss of heterozygosity
PAP	pulmonary artery pressure
GADD45 FCS	growth arrest and DNA damage-inducible 45 Fetal Calf Serum
