# Supplementary material for: Smooth Muscle Phenotype in Idiopathic Pulmonary Hypertension: Hyper-Proliferative but not Cancerous
Source: Int J Mol Sci. 2019 Jul 22;20(14):3575. doi: 10.3390/ijms20143575 (PMC6679125; doi:10.3390/ijms20143575)
Supplement: Supplementary file 1 [file ijms-20-03575-s001.pdf]

International Journal of
Molecular Sciences

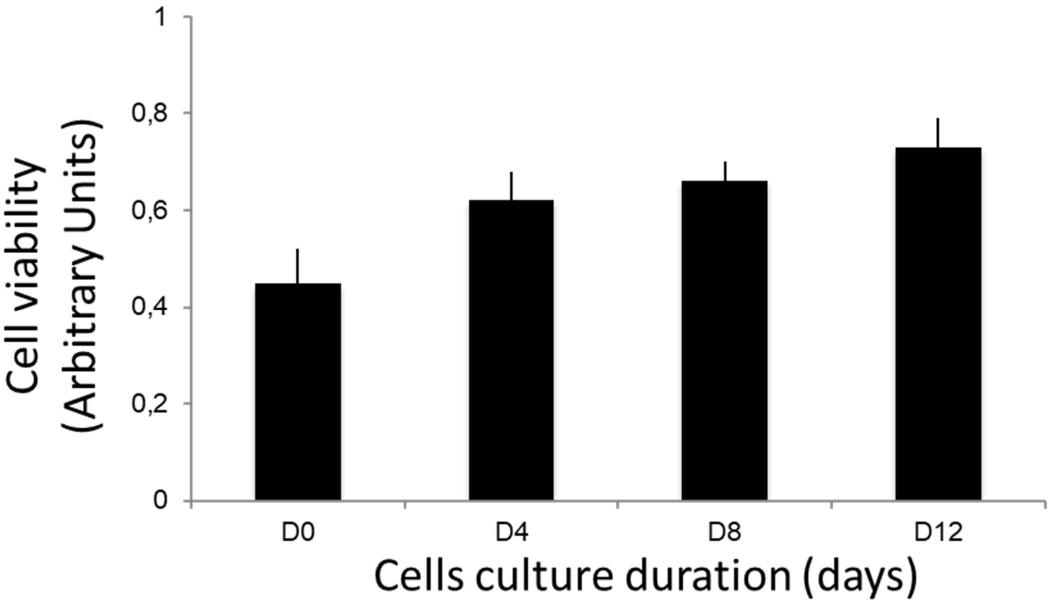

Figure S1. Cancer cells display self-sufficient proliferation. Human breast cancer cells line MDA-MB-231 were seeded in 12-well plates in 1% FCS/DMEM at a density of 10^5 cells/well and allowed to adhere for 24 h. The medium was subsequently removed, and the cells were subjected to serum-free DMEM for 4, 8 and 12 days. At the end of each period of incubation, the cells were dissociated by trypsin and counted by using a Muse Cell Analyzer following the manufacturer's instructions (Merck).

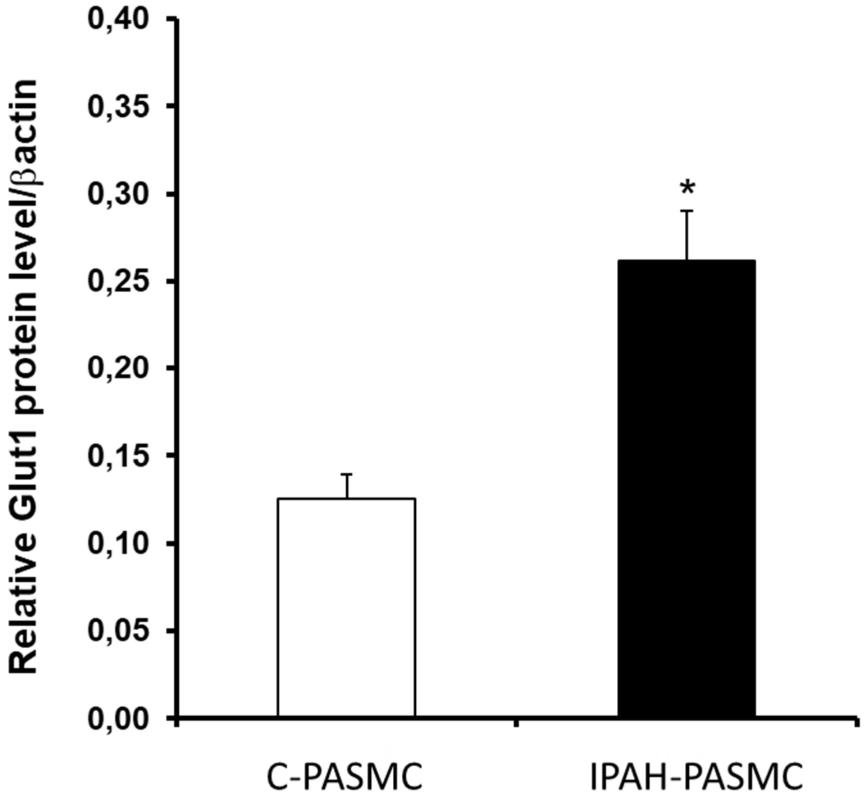

Figure S2. Overexpression of glucose transporter Glut1 in IPAH-PASMC. PASMC from controls and from IPAH patients were lysed as described in method section. Forty µg proteins were separated in 10% sodium dodecyl sulfate polyacrylamide gel electrophoresis and transferred to nitrocellulose membranes. Glucose transporter (Glut1) was detected using rabbit polyclonal anti-Glut1 (ab15309, abcam, Paris, France). Then HRP conjugated anti-rabbit secondary antibody was used at a dilution of 1:10000 (Calbiochem, Fontenay-Sous-Bois, France). Immunoreactive bands were visualized using chemiluminescence (ECL) (GE Healthcare) on a Bio-Rad Fluoro-S-Max Chemidoc system. A polyclonal antibody against β-actin (diluted 1:3000; Sigma Aldrich) served as the internal loading control. Densitometric quantification of the immunoblot bands was performed using Bio-Rad Quantity One software. Glut1 protein level was higher in IPAH than in controls PASMCs, * $p < 0.01$.

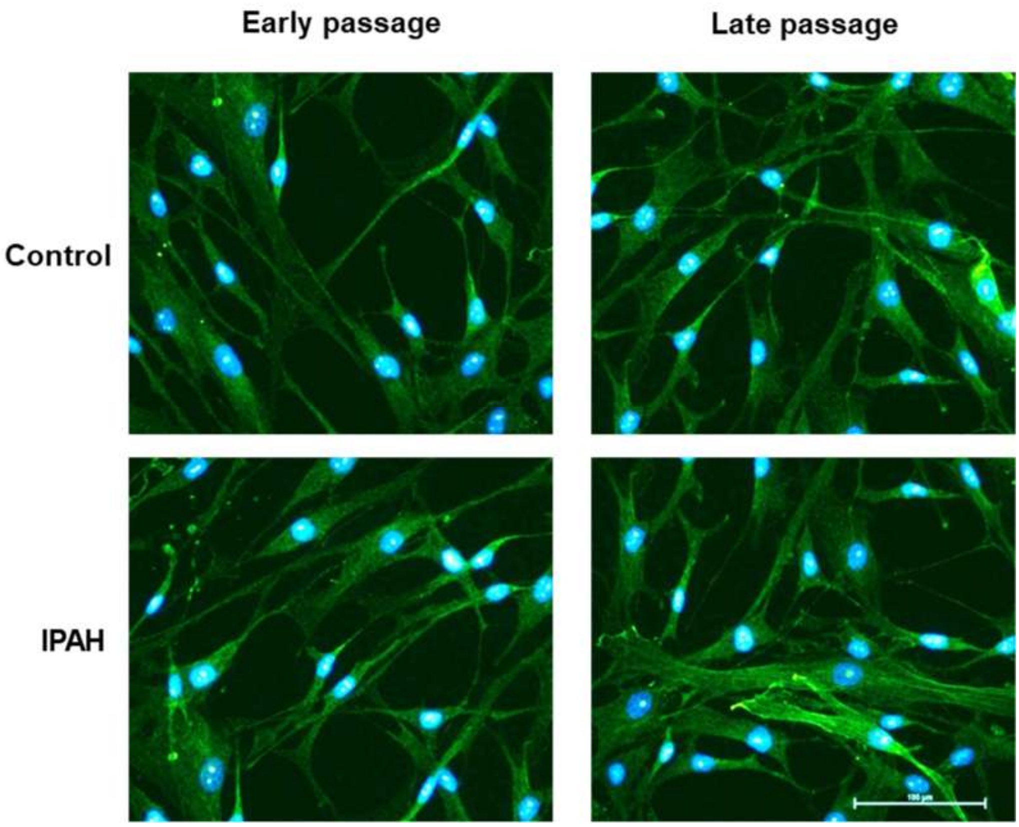

Figure S3. Immunofluorescence microscopy revealed stable expression of the smooth muscle cell marker SM22 by cultured control and IPAH PASMCs from early (P3) to late (P8) passages. Pulmonary artery smooth muscle cells (PASMC) from control and from IPAH patients were seeded in 6 well plates at a density of 1 × 105 cells/well and allowed to grow in the same medium with 10%FCS for 24 h. The cells were washed twice with ice-cold (PBS), and fixed with methanol. After blocking with PBS containing 1% (w/v) bovine serum albumin (BSA) and 0.1% (v/v) Tween 20, the cells were incubated at 4°C overnight with anti-human SM22 antibody (1/200, Abcam). Fuorescein-conjugated IgG (Vector Laboratories, Inc.) was used as the secondary antibody. Nuclear counterstaining using DAPI; Sigma-Aldrich) was performed according to the manufacturer's recommendations.
